# Musculoskeletal Disorders Related to Upper Limb Disability after One-Year Lung Cancer Resection

**DOI:** 10.3390/cancers16122279

**Published:** 2024-06-19

**Authors:** Javier Martín Núñez, Julia Raya Benítez, Florencio Quero Valenzuela, Andrés Calvache Mateo, Alba Navas Otero, Alejandro Heredia Ciuró, Marie Carmen Valenza

**Affiliations:** 1 Department of Physiotherapy, Faculty of Health Sciences, University of Granada, 18071 Granada, Spain; javimn@ugr.es (J.M.N.); andrescalvache@ugr.es (A.C.M.); albanavas@ugr.es (A.N.O.); cvalenza@ugr.es (M.C.V.); 2 Department of Nursing, Faculty of Health Sciences, University of Granada, 18071 Granada, Spain; juliarb@ugr.es; 3 Thoracic Surgery Department, Hospital Virgen de las Nieves de Granda, 18014 Granada, Spain

**Keywords:** lung cancer, pulmonary surgical procedures, musculoskeletal disorders, upper limb, disability

## Abstract

**Simple Summary:**

Lung resection is the main curative treatment for lung cancer, but it can cause several tissue and organ disorders. While cardiovascular, pulmonary, and muscular disturbances post-surgery have been studied, long-term upper limb impairment has not been extensively explored despite its impact on patient independence. This study aimed to characterize upper limb impairment in survivors of lung cancer one year after surgery. In an observational trial, 76 patients with lung cancer who had undergone surgery were compared to 74 healthy controls. Our results revealed significant differences in active shoulder mobility, pain hypersensitivity, neural tissue mechanosensitivity, and upper limb exercise capacity. These results indicate that survivors of lung cancer experience significant upper limb musculoskeletal disorders and functional impairment one year post-resection, which can limit their functionality and quality of life.

**Abstract:**

Lung resection represents the main curative treatment in lung cancer; however, this surgical process leads to several disorders in tissues and organs. Previous studies have reported cardiovascular, pulmonary, and muscular disturbances that affect the functional capacity of these patients in the short, mid, and long term. However, upper limb impairment has been scarcely explored in the long term, despite the relevance in the independence of the patients. The aim of this study was to characterize the upper limb impairment in survivors of lung cancer one year after pulmonary resection. In this observational trial, patients who underwent lung cancer surgery were compared to control, healthy subjects matched by age and gender. Upper limb musculoskeletal disorders (shoulder range of motion, pain pressure threshold, nerve-related symptoms) and functional capacity (upper limb exercise capacity) were evaluated one-year post-surgery. A total of 76 survivors of lung cancer and 74 healthy subjects were included in the study. Significant differences between groups were found for active shoulder mobility (*p* < 0.05), widespread hypersensitivity to mechanical pain (*p* < 0.001), mechanosensitivity of the neural tissue (*p* < 0.001), and upper limb exercise capacity (*p* < 0.001). Patients who undergo lung cancer surgery show upper limb musculoskeletal disorders and upper limb functional impairment after a one-year lung resection. This clinical condition could limit the functionality and quality of life of patients with lung cancer.

## 1. Introduction

Pulmonary resection represents the main treatment for patients with lung cancer [1]. This approach consists of removing the tumor of lung tissue, preventing it from growing into adjacent tissues [2]. Although in recent years pulmonary resection procedures have undergone a major evolution with less invasive and quick techniques [3], they are still associated with a high incidence of postoperative complications [4].

Patients with lung cancer accept complications as a possible risk of the surgery process; however, they are not expected to have disability issues [5]. The disability rate in these patients has become a major health problem due to the current high prevalence accompanying increased survival in recent years. In this sense, survivors of lung cancer reported that the causes of disability often do not resolve after lung resection, and disability issues remain between 3 and 6 months and up to 5 years after lung surgery [6]. For these reasons, achieving a functional lifestyle and a high quality of life has become one of the main long-term therapeutic goals [7].

Pain and shoulder dysfunction are the most common disability issues after lung resection, affecting the quality of life of patients with lung cancer [8]. Shoulder impairment has been widely explored [9] and has been associated with several upper limb musculoskeletal disorders [10]. The most reported have been shoulder pain [11], limited range of motion, and functional disturbances from the upper limbs, including limitations in the ability to perform activities of daily living, lifting above shoulder level, or performing sports activities [12].

Previous studies have observed that upper limb musculoskeletal disorders are still present one month after lung resection [10], being reported in more than 20% of patients with lung cancer [13]. However, there are uncertain results when these sequelae are studied after six months of surgery [14,15,16]. Considering the close relationship reported between upper limb impairment and disability in survivors of lung cancer, this lack of knowledge is worrisome due to the remarkable rates of disability in long-term survivors of lung cancer [17].

In this line, no previous studies have explored upper limb musculoskeletal disorders in patients who underwent lung cancer surgery in the long term. Therefore, the aim of this study was to explore and characterize the upper limb musculoskeletal disorders suffered by survivors of lung cancer one year after lung resection.

## 2. Materials and Methods

We conducted a single-blind observational study in which patients undergoing lung resection were compared to healthy subjects matched by age and gender. Sample size calculation was performed with G*Power 3.1.9.2 software based on the data of a previous study published by the research group [10], adopting statistical power of 95%, α = 0.05, and allocation ratio between groups = 1, resulting in a calculated sample size of 146 (73 per group). However, 87 participants per group were recruited to allow for a dropout rate of 20%.

Patients with lung cancer were recruited from the Thoracic Surgery Service of the “Hospital Universitario Virgen de las Nieves de Granada” (HVN) between October 2019 and February 2020. Healthy subjects were recruited from the general community through announcements at the University of Granada (Spain). Assessors were blinded before evaluations were scheduled and other team members booked the patients’ visits. Informed consent was obtained from all included participants. This study protocol was developed according to STROBE guidelines and was reviewed and approved by the Granada Provincial Research Ethics Committee (Spain), with the code: FISIOLUNG: 12 March 2018 [18].

The inclusion criteria included the following: survivors of lung cancer aged 18–80 years who had undergone resection surgery one year ago, were informed about the study purpose, and signed the informed consent. Exclusion criteria included the following: cognitive impairment, mental instability, orthopedic pathologies that limited the test performance or neurologic pathologies that could limit the patient’s comprehension or expression or could make it difficult to perform the physical assessment.

Data collection was carried out one-year after surgery, consistently conducted by pre-trained investigators. Inclusion criteria were confirmed, and a structured interview and initial assessment were conducted. Pertinent medical history data including anthropometric measurements, comorbidities (evaluated using the Charlson comorbidities index) [19], length of stay, surgical duration, and spirometry values (forced expiratory volume in the first second (FEV-1)) [20] were retrieved.

The main outcomes included upper limb musculoskeletal disorders (shoulder range of motion, pain pressure threshold, nerve-related symptoms) and functional capacity, which was studied with upper limb exercise capacity.

Active shoulder mobility was evaluated using a laser-guided digital goniometer (Halo, Halo Medical Devices, Subiaco, Western Australia) and expressed in degrees [21]. The assessment included the following movements: flexion, extension, adduction, abduction, and internal and external rotations of both arms. Patients stood while the researcher instructed and supervised the execution of these movements, ensuring correct posture and correcting any compensations. The reliability of this tool has demonstrated a high accuracy (intra-class correlation coefficient > 0.90) [22].

Widespread hypersensitivity to mechanical pain was measured by assessing pain tolerance thresholds to pressure (PPT) [23], which were measured with a digital algometer (Model Mark-10 M3-20 Series, Copiague, NY, USA). This algometer consisted of a circular platform with an area of 1 cm^2^ rubber tip and a range from 0 to 12 kg. The subjects were instructed to press a switch immediately when the sensation of pain was the highest tolerable. The mean of the three trials was calculated for the main analysis. Algometry measuring PPT exhibited high intra- and inter-rater reliability {intraclass correlation coefficient = 0.80–0.97)} [24,25]. The PPTs were assessed bilaterally in random order at a local point of the anterior deltoid muscle and a distal pain-free point on the flexor carpi longus muscle, to determine the widespread pressure hypersensitivity [26,27].

Neurodynamics testing was used to assess neural tolerance to movement. Passive neurodynamics tests were performed according to the process described by Butler [28], in nerves which have been used in previous thoracic cancer studies [29]: median, ulnar, and radial nerves. Neurodynamics tests were conducted bilaterally, following a standardized sequence until reaching the end of the range or symptom reproduction. Shoulder and elbow range of motion (ROM) at the conclusion of the tests were measured using a standard goniometer based on a previously reported procedure [30].

The unsupported upper limb exercise (UULEX) test, devised by Takahashi et al. [31], is an incremental test to measure peak unsupported arm exercise capacity. Participants lift a barbell from their lap to the highest point they can reach in levels, continuing until exhaustion. The total score is determined by the total duration of the test in seconds. Self-reported dyspnea and upper limb fatigue were evaluated using a modified version of the Borg scale [32], with a minimum clinically important difference (MCID) set at 1 score.

### Statistical Analysis

The statistical package SPSS version 27.0 (International Business Machines, Armonk, NY, USA) was used to analyze the data obtained. Descriptive statistics (mean ± SD) or percentages (%) were used to describe sample baseline characteristics. The Kolmogorov–Smirnov test was performed to assess continuous data normality, prior to statistical analysis. T-Student’s test for unpaired samples was employed to perform the data analysis, comparing test results between case and control subjects. A *p* value of less than 0.05 was considered statistically significant. A 95% confidence interval was used for statistical analysis.

## 3. Results

**Figure 1:**
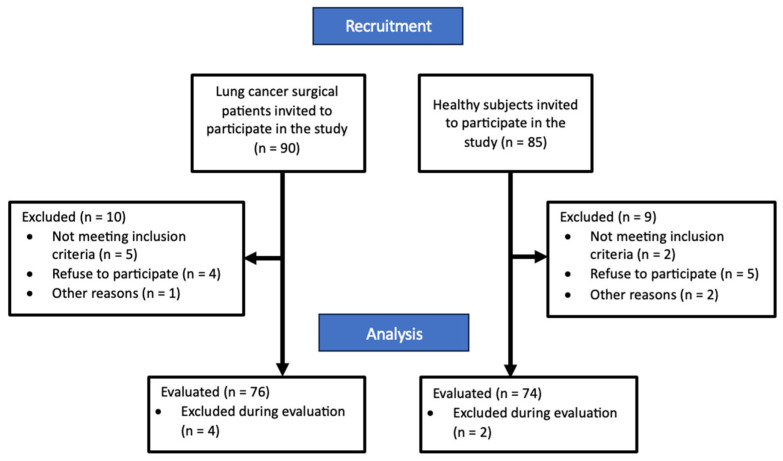
Flow diagram for distribution of participants.

A total of 90 potential patients with lung cancer were initially invited to participate in the study, 80 were considered eligible based on meeting inclusion criteria and consented to participate. A total of four patients with lung cancer and two healthy subjects were excluded during the assessment because drop out or physical limitations. Finally, 76 participants completed the evaluation process. Additionally, 85 healthy control subjects, matched for age and gender, were recruited, with 76 consenting and meeting inclusion criteria, and 74 completing the evaluation. Participant distribution is illustrated in Figure 1.

The baseline characteristics of the sample are presented in Table 1. The mean age of the groups was 58.98 ± 13.34 in the lung resection group and 58.75 ± 11.01 in the control group (*p* = 0.856). Both groups presented a similar sex distribution (84.3%; 85%; *p* = 0.785), and similar BMI (*p* = 0.614). There were no significant differences in the associated comorbidities according to the Charlson index (*p* = 0.705) and FEV-1 values (*p* = 0.764). The duration of the lung resection was around 156.78 min, and the hospital stay 6.82 ± 2.03 days.

Table 2 shows the results of the between-groups comparison of the upper limb musculoskeletal disorders. Active shoulder range of motion showed significant differences in favor of the control group for flexion of the intervened side (*p* = 0.033), and the abduction of the no intervened side (*p* < 0.001) and external rotation (*p* < 0.001). Adduction and internal rotation of both sides also showed significant differences (*p* < 0.05).

The abduction of the intervened side and external rotation were minor in the lung resection group; however, no significant differences were found. The flexion of the no intervened side, and extension of both upper limbs were similar between groups.

The assessment of the pain pressure threshold showed significant differences in the intervened side (*p* = 0.004) and no intervened side (*p* < 0.001) anterior deltoid muscle, and both flexor carpi longus muscles (*p* < 0.001), with higher values for the control groups.

Significant differences were also found during neurodynamics testing. The median nerve showed significant differences for the intervened (*p* = 0.001) and no intervened (*p* < 0.001) upper limb, in a similar way to the radial nerve (*p* < 0.001) (*p* = 0.007). The ulnar nerve presented significant differences in the no intervened side to surgery (*p* = 0.007), but no significant differences appeared on the intervened side, although the control group showed a wider range of motion.

Table 3 shows the results of the UULEX test. The groups showed significant differences in the achieved level (*p* < 0.001) and the spent time (*p* < 0.001), in favor of the control group. However, the fatigue and dyspnea reported at the end of the test did not show significant differences (*p* = 0.194 and *p* = 0.694, respectively).

## 4. Discussion

This study aimed to characterize upper musculoskeletal impairment in survivors of lung cancer one year after lung resection. Our results showed the presence of musculoskeletal disorders in the upper limbs and limited functional capacity one year after undergoing pulmonary resection. These results were in line with previous studies that reported post-thoracotomy painful syndrome even one year after VATs lobectomy [33].

The sample of subjects included in this study was representative of the general population undergoing lung resection, with similar sociodemographic characteristics [34,35]. In a similar way to our sample, the study by Ren D et al. [36], which observed the occurrence of ipsilateral shoulder pain after thoracoscopy, presented a sample with similar age, BMI, and duration of surgery.

Our results showed that the active range of motion of the shoulder complex was reduced in patients who had undergone pulmonary resection one year before. Consistent with our results, previous studies [10] observed a bilateral reduction in active shoulder range of motion after pulmonary resection. The study of Miranda APB et al. [8] also observed a bilateral reduction in shoulder range of motion in patients who underwent thoracotomy, which they attributed to the patient’s position during surgery on the ipsilateral side, and to the pressure of the patient’s weight on the contralateral side. However, they did not follow up on these musculoskeletal disorders and their consequent evolution in the long term.

Previous studies in similar populations show similar results to ours in algometry after medical treatment [37]. The study by Rodriguez-Torres J et al. [10] also observed similar results to ours, in which patients with lung cancer who received a lung resection presented a widespread hypersensitivity to mechanical pain at mid-term.

With regard to neurodynamic tests, we have not found studies that assess the mechanosensitivity of the neural tissue in this population, but studies in similar populations [38] also found results consistent with ours, showing a decrease in the ROM of these tests as a protective mechanism [38].

The fact that the algometric and neurodynamic pain tolerance results show statistically significant differences, with worse results in the lung resection group, may be due to problems with mechanosensitivity and/or central sensitivity [39]. Previous studies [40] have highlighted that acute thoracic pain after surgery could affect neuronal plasticity and be a good predictor of chronic pain.

In terms of upper extremity functional capacity, as assessed by the UULEX test, our results showed lower exercise capacity in the pulmonary resection group at one year. To the best of our knowledge, this is the first study to evaluate functionality in patients with pulmonary resection after one year, although previous studies [41] have found similar results in the first month after surgery. A decrease in functional capacity and muscle endurance after surgery has also been observed in similar populations, such as after breast cancer or cardiac surgeries [42,43]. However, these studies did not present long-term results.

The functional capacity assessment was carried out using the UULEX test and not a hand-held dynamometer, which is more widely used, since the UULEX is considered a test with better capacity and greater relevance for reflecting the individual’s performance in activities of daily living [31]. Both tests show a moderate to high correlation, which is probably due to the fact that the muscle activity required to complete them is similar (mainly elbow and shoulder flexion) [44].

### 4.1. Limitations

Our study has several limitations to be mentioned. The sample size is limited because of the difficulty in recruiting subjects who met the selection criteria; however, previous studies have used similar sample sizes in this population [45]. In addition, in terms of study design, a prospective study with preoperative and postoperative evaluation may have been more rewarding, allowing each patient to be in his or her own control. However, the control group was carefully selected, matching age and sex with the group of patients evaluated and avoiding other pathologies, so as not to influence the results or introduce a possible source of bias. Finally, some characteristics of the study sample, including the age range and physical activity levels, could disturb the differences found between the groups. Future studies with multivariate regression analyses are necessary to observe the influence of these aspects on the upper limb musculoskeletal disorders presented by patients with lung cancer in the long term.

### 4.2. Clinical Implications

The study of these long-term alterations is an important factor to consider. Knowledge of the risk of long-term disability is one of the determining factors in the surgical decision [46]. Additionally, long-term upper limb impairment is a key factor in the patient’s functionality, as it limits the activities of daily living [12], reducing the quality of life of these patients [47]. It is also important to define the possibility of presenting central and peripheral sensitization, as this implies greater difficulty in the rehabilitation approach [48]. For these reasons, the significance of this study in reducing the comorbidity of these patients, making easier their recovery after the intervention, and preventing disability one year after the surgery is highlighted.

Moreover, knowing musculoskeletal disorders related to upper limb disability after one-year lung cancer resection could also make the design of future rehabilitation interventions easier to manage the musculoskeletal disorders that disturb the quality of life of these patients in the long term.

## 5. Conclusions

Patients with lung cancer who suffer a pulmonary resection show upper limb musculoskeletal disorders and functional impairment after one-year lung resection, exposing the presence of a limited active shoulder range of motion, widespread hypersensitivity to mechanical pain, mechanosensitivity of the neural tissue, and low exercise capacity, which could be altering the functionality of the patients and their quality of life.

Future studies with a longer follow-up period could help to obtain more precise data on the chronic physical impairment suffered by these patients. Future research could also be proposed with interventions that approach the mentioned alterations, thus improving the quality of life of these patients.

## Figures and Tables

**Table 1:** Baseline characteristics of the sample.

Variables	Resection Group (n = 76)	Control Group (n = 74)	*p*
Sex distribution (% male)	64 (84.3%)	63 (85%)	0.785
Age (years)	58.98 ± 13.34	58.75 ± 11.01	0.856
BMI (kg/m^2^)	27.58 ± 12.5	29.35 ± 15.47	0.614
Charlson Index	4.55 ± 2.51	4.47 ± 1.99	0.705
FEV1%	80.44 ± 23.63	81.02 ± 24.55	0.764
Surgery duration (minutes)	156.78 ± 24.3	-	-
Length of stay	6.82 ± 2.03	-	-

Variables were expressed as mean ± Standard Deviation or Percentage (%). BMI: body mass index; FEV1%: Forced expiratory volume in the first second.

**Table 2:** Musculoskeletal disorders in patients with lung resected cancer and healthy control subjects.

	Resection Group (n = 76)	Control Group (n = 74)	*p*
AROM			
Flexion (°)			
Intervened Side	152.42 ± 3.60	159.36 ± 16.58	0.033 *
No Intervened Side	166.00 ± 12.01	162.52 ± 12.20	0.252
Extension (°)			
Intervened Side	50.00 ± 6.88	52.16 ± 9.64	0.300
No Intervened Side	51.50 ± 8.45	50.60 ± 8.86	0.679
Abduction (°)			
Intervened Side	159.14 ± 34.80	165.84 ± 17.44	0.261
No Intervened Side	166.28 ± 15.00	180.00 ± 0.00	<0.001 **
Adduction (°)			
Intervened Side	18.85 ± 3.41	36.52 ± 24.45	<0.001 **
No Intervened Side	18.00 ± 3.12	31.08 ± 20.52	0.003 *
Internal Rotation (°)			
Intervened Side	37.42 ± 7.90	51.92 ± 20.91	0.001 *
No Intervened Side	34.00 ± 7.64	52.32 ± 18.23	<0.001 **
External Rotation (°)			
Intervened Side	77.14 ± 6.87	81.56 ± 12.79	0.095
No Intervened Side	72.33 ± 9.09	83.20 ± 9.31	<0.001 **
Pain Pressure Threshold			
Deltoid (kg)			
Intervened Side	4.19 ± 3.24	5.78 ± 2.13	0.004 *
No Intervened Side	3.63 ± 2.76	5.94 ± 2.12	<0.001 **
Carpal Long flexor muscle (kg)			
Intervened Side	4.44 ± 3.46	6.68 ± 2.86	<0.001 **
No Intervened Side	4.26 ± 3.43	6.79 ± 2.83	<0.001 **
Neurodynamic test			
Median (°)			
Intervened Side	122.41 ± 36.07	141.04 ± 30.41	0.001 *
No Intervened Side	123.45 ± 28.51	141.30 ± 27.36	<0.001 **
Ulnar (°)			
Intervened Side	114.52 ± 17.51	119.05 ± 42.98	0.421
No Intervened Side	123.63 ± 22.66	137.71 ± 35.83	0.007 *
Radial (°)			
Intervened Side	75.47 ± 24.24	95.48 ± 38.63	<0.001 **
No Intervened Side	70.98 ± 30.72	84.45 ± 27.48	0.007 *

Variables were expressed as mean ± standard deviation. Significant results are expressed as ** *p* < 0.001. AROM: active range of motion; °: grades; kg: kilograms.

**Table 3:** Functional capacity of patients with lung resected cancer and healthy control subjects.

	Resection Group (n = 76)	Control Group (n = 74)	*p*
UULEX level	3.94 ± 2.88	6.81 ± 1.61	<0.001 **
UULEX weight (grames)	505.88 ± 631.01	454.77 ± 667.37	0.649
UULEX time (seconds)	362.11 ± 272.82	529.43 ± 157.79	<0.001 **
UULEX Fatigue Post	4.88 ± 2.34	4.24 ± 3.27	0.194
UULEX Dyspnea Post	2.34 ± 1.18	2.24 ± 1.17	0.694

Variables were expressed as mean ± standard deviation. Significant results are expressed as * *p* < 0.05 and ** *p* < 0.001. UULEX: unsupported upper limb exercise test.

## Data Availability

Data is contained within the article.
